# Evaluation of Gastrointestinal Leakage in Multiple Enteric Inflammation Models in Chickens

**DOI:** 10.3389/fvets.2015.00066

**Published:** 2015-12-14

**Authors:** Vivek A. Kuttappan, Eduardo A. Vicuña, Juan D. Latorre, Amanda D. Wolfenden, Guillermo I. Téllez, Billy M. Hargis, Lisa R. Bielke

**Affiliations:** ^1^Department of Poultry Science, University of Arkansas, Fayetteville, AR, USA

**Keywords:** dextran sodium sulfate, serum FITC-d, rye-based diet, enteric inflammation, feed restriction

## Abstract

Enteric inflammation models can help researchers’ study methods to improve health and performance and evaluate various growth promoters and dietary formulations targeted to improve performance in poultry. Oral administration of fluorescein isothiocyanate-dextran (FITC-d; 3–5 kDa) and its pericellular mucosal epithelial leakage are an established marker to evaluate enteric inflammation in multiple species. The present study evaluated different methods to induce gut inflammation in poultry based on FITC-d leakage. Four independent experiments were completed with different inflammation treatment groups, and serum FITC-d and/or retention of FITC-d in GI tract were determined. In experiment 1 (*n* = 10 birds/treatment, broilers, processed at 14 days), groups included control (CON), dextran sodium sulfate (DSS; drinking water at 0.75%) and feed restriction (FRS; 24 h before processing). Experiment 2 (*n* = 14 birds/treatment, leghorns, processed at 7 days) included CON, DSS, FRS, and rye-based diet (RBD). In experiments 3 and 4 (*n* = 15 birds/treatment, broilers, processed at 7 days), groups were CON, DSS, high fat diet (HFD), FRS, and RBD. In all experiments, FRS and RBD treatments showed significantly higher serum FITC-d levels compared to the respective CON. This indicates that FRS and RBD results in disruption of the intact barrier of the gastrointestinal tract (GIT), resulting in increased gut permeability. DSS and HFD groups showed elevation of serum FITC-d levels although the magnitude of difference from respective CON was inconsistent between experiments. FRS was the only treatment which consistently showed elevated retention of FITC-d in GIT in all experiments. The results from present studies showed that FRS and RBD, based on serum FITC-d levels, can be robust models to induce gut leakage in birds in different age and species/strains.

## Introduction

It is well known that antibiotic growth promoters (AGP) can improve production performance in birds (1) although the exact mode of action is still not completely understood. According to (2), a major hypothesis about the action of AGP is through the reduction of innate inflammatory response in the gastrointestinal tract (GIT) of birds. Now that the use of AGP is discouraged in food animals, and worldwide, it is time to identify growth promoters which are as effective or even better and at the same time safer than AGP.

In addition to its role in endocrine and paracrine hormones’ production, nutrient permeability, water and electrolyte exchange, and digestion, the intestinal mucosa is an important barrier for protecting animals against both commensal and pathogenic microorganisms and other insults (3–5). The intestinal first line of defense is composed of the mucus layer and epithelium (6). It has been proposed that intestinal epithelial cell defenses are essential to prevent inflammation, for example, by offering protection against microbial pathogens and oxidative stresses (7). If the intestinal barrier is damaged and becomes non-selectively permeable, the submucosa and deeper layers will be subjected to continuous exposure to antigenic molecules from food and microorganisms, causing inflammatory conditions (8).

Dextran sodium sulfate (DSS) is a heparin-like polysaccharide (9) which can cause disruption of the epithelial lining in the GIT of poultry (10). In addition, our laboratory has already used the gut inflammation induced with DSS to identify various markers to measure gut health.

Fluorescein isothiocyanate-dextran is an established method to measure paracellular leakage in rodent enteric inflammation models (11–13). Since increased mucosal permeability is the first step in the cascade of enteric inflammation, fluorescein isothiocyanate-dextran (FITC-d) model to identify gut leakage would be an effective way to detect the efficiency of various alternative growth promoters, quite early, and predict their beneficial effect on growth and production performance. Studies in our laboratory thus far have shown that oral administration of FITC-d, its leakage to circulation through disrupted epithelial lining of GIT, and subsequent measurement of FITC-d levels in serum is effective in evaluating gut leakage in birds (14, 15). The main use of this marker is to determine the effect of various growth promoters on gut health in birds.

Although DSS produces enteric inflammation in birds, our previous studies suggest that birds were more sensitive to DSS (10) compared to rodents, and achieving acceptable levels of toxicity and lesions was very narrow. Thus, other methods of induction of mucosal permeability in poultry were tested and compared with the previously established DSS model. Alternatives for DSS were feed restriction (FRS) and dietary models, such as rye-based diet (RBD) and high fat diet (HFD). A major advantage to these alternatives was the reduced risk of toxicity and severe illness to birds. At the same time, these alternatives represented various real-world scenarios which can reduced in production performance in poultry.

Feed restriction has been historically used as a way to maintain appropriate body weight of parent stock in meat-type chickens to reduce lameness and health risks, as well as improve fertility rates (16, 17). However, FRS has also been shown to increase enteric permeability, translocation of enteric bacteria to various organs (14), and could make birds more susceptible to various disease conditions related to translocation of pathogens from the GIT to systemic circulation. In addition, some regions of the world use alternate feed stuffs such as rye in place of corn. But, high levels of non-starch polysaccharides (NSP) in rye result in increased digesta viscosity as well as other associated gut health problems, thereby increasing the chance of necrotic enteritis (18–20). Furthermore, inclusion of higher levels of fat in poultry diet has been a common practice that increases growth rate; however, some of studies in mice have shown that this could also produce enteric health problems (21). Based on these facts, the main objective of the present studies was to compare the effect of DSS, FRS, HFD, and RBD on enteric leakage in chickens, measured using serum FITC-d model.

## Materials and Methods

### Experimental Design

Four independent experiments were conducted, and each used different combinations of diets (Table 1), as well as other treatments such as FRS (for 24 h before processing) and DSS (MW 40,000; Alfa Aesar, Ward Hill, MA, USA) administered 0.75% in drinking water for 3 days before processing, as given below. Different diets used in the study were basal, RBD, and HFD, of which all met or exceeded NRC requirements (22). In all experiments, birds were administered FITC-d (MW 3,000–5,000 Da; Sigma-Aldrich Co., St. Louis, MO, USA) by oral gavage, 2.5 h before processing, to determine levels of mucosal leakage by evaluating serum FITC-d and the level of FITC-d retained in different regions of GI tract. Birds were humanely killed by inhalation of carbon dioxide gas prior to the collection of blood and GIT samples. All studies were conducted in accordance with protocols approved by the University of Arkansas Institutional Animal Care and Use Committee.

**Table 1:** **Ingredients of the feed formulations used in the study**.

	CON^a^	HFD^b^	RBD^c^
**Ingredients (%)**
Corn	56.59	53.57	–
Rye	–	–	58.19
Soybean meal	35.74	35.13	31.16
Vegetable oil	3.29	6.60	6.29
Dicalcium phosphate	1.81	1.87	1.79
Calcium carbonate	1.12	0.98	1.05
Salt	0.38	0.52	0.38
dl-Methionine	0.31	0.34	0.35
Vitamin premix^d^	0.20	0.20	0.20
l-Lysine HCL	0.19	0.30	0.22
Choline chloride 60%	0.10	0.20	0.10
Mineral premix^e^	0.10	0.10	0.10
Threonine	0.06	0.16	0.08
Antioxidant^f^	0.02	0.02	0.02
Mold propionic acid	0.05	0.05	0.05
Total	100	100	100
**Calculated analysis**
ME (kcal/kg)	3,035	3,191	2,850
CP (%)	21.7	22.1	22.4
Lys (%)	1.32	1.35	1.32
Met (%)	0.63	0.64	0.64
Met + Cys (%)	0.98	0.99	0.98
Thr (%)	0.86	0.91	0.86
Trp (%)	0.25	0.28	0.3
Total calcium (%)	0.9	0.9	0.9
Available phosphorus (%)	0.45	0.45	0.45
Sodium (%)	0.16	0.21	0.16

*^a^CON, control*.

*^b^HFD, high fat diet*.

*^c^RBD, rye-based diet*.

*^d^Vitamin premix (per 1,000 kg): vitamin A, 20,000,000 IU; vitamin D3, 6,000,000 IU; vitamin E, 75,000 IU; vitamin K3, 9 g; thiamine, 3 g; riboflavin, 8 g; pantothenic acid, 18 g; niacin, 60 g; pyridoxine, 5 g; folic acid, 2 g; biotin, 0.2 g; cyanocobalamin, 16 mg; and ascorbic acid, 200 g (Nutra Blend LLC, Neosho, MO, USA)*.

*^e^Mineral premix (per 1,000 kg): manganese, 120 g; zinc, 100 g; iron, 120 g; copper, 10–15 g; iodine, 0.7 g; selenium, 0.4 g; and cobalt, 0.2 g (Nutra Blend LLC)*.

*^f^Ethoxyquin*.

In experiment 1, 30-day-old broiler chicks were randomly assigned to one of the three treatment groups (*n* = 10/group), control (CON), FRS, or DSS. All birds were provided *ad libitum* with the basal diet (Table 1) until 13 days. Twenty-four hours prior to the end of the experiment, feed was removed from FRS through the end of the experiment. On day 14, all birds were given an oral gavage of FITC-d (2.2 mg/mL/bird) 2.5 h before they were processed. Blood and tissue samples from duodenum, ileum, and cecum were collected from each bird. Levels of FITC-d in serum and GI tract were determined as explained below.

Day of hatch Leghorns (*n* = 56) was used for experiment 2 and was randomly assigned to CON, DSS, FRS, and RBD (*n* = 14/treatment). In this experiment, all treatment groups except RBD were given basal diet, while RBD was given a RBD (Table 1), and feed removed from FRS 24 h prior to end of the experiment. All birds were processed on day 7 after oral gavage with FITC-d (2.2 mg/bird) 2.5 h before processing. Blood and GIT tissue (duodenum and cecum) samples were collected for determination of enteric inflammation.

Experiments 3 and 4 had the same experimental design but were conducted independently. For both experiment, 75-day-old broiler chicks were randomly assigned to CON, DSS, FRS, RBD, or HFD group (*n* = 15 birds/treatment). All groups except RBD and HFD were given basal diet throughout the trail while RBD and HFD were given RBD as well as HFDs, respectively (Table 1). As in previous experiments, feed was removed from FRS 24 h prior to the end. On day 7, all birds were given oral gavage with FITC-d (2.2 mg/bird), and serum level and GIT retention (duodenum and cecum) of FITC-d were determined.

### Determination of Serum FITC-d Levels

Serum level of FITC-d, a measurement of enteric inflammation and mucosal permeability, was determined as explained by Kuttappan et al. (14). After humane slaughter of birds, femoral artery was severed to collect blood and allowed to clot under room temperature for 3 h. Then, the samples were centrifuged at 500 × *g* for 15 min, and serum samples were collected. The serum samples were then diluted in phosphate buffer saline (1:1), and fluorescence was measured at 485 nm excitation and 528 nm emission (Synergy HT, multimode microplate reader, BioTek Instruments, Inc., VT, USA). Levels of fluorescence in the samples were converted to respective FITC-d microgram per milliliter of serum based on a calculated standard curve previously obtained from known levels of FITC-d.

### Level of FITC-d Retained in Gastrointestinal Tract Tissue

Amount of FITC-d retained in different regions of GI tract were determined using the method suggested by Kuttappan et al. (14) and Vicuña et al. (15). For this, 2.5-cm-long tissue sections were collected from the descending duodenum, ileum immediately proximal to the Meckel’s diverticulum, and a single entire cecum (opened at both ends). These samples were cleaned by flushing with Hanks buffered salt solution. After cleaning, samples were gently mopped to remove excess fluid, weighed, and dropped in tubes containing 10 mL Hanks buffer with glutamine (0.3 g/L) and antimicrobial agents (penicillin 100 U/mL, streptomycin 0.01 mg/mL, and amphotericin B 0.25 μg/mL). The tubes were incubated at 42°C for 2 h, and the FITC-d released to the buffer from the tissue was determined and reported as microgram per gram of the respective tissue.

### Statistical Analysis

Data were analyzed using ANOVA (SAS 9.3, SAS Institute Inc., Cary, NC, USA) considering individual birds as experimental units for all experiments. Means were separated using Duncan’s significant different test at *p* < 0.05. The serum FITC-d data showed occasional, but random outliers, <+2 SD from group mean, which were not representative of the respective groups similar to the reports by Kuttappan et al. (14) and Vicuña et al. (15). It is clear that these outliers were not related to treatments administered; however, the reasons for the occurrence of these erratic outliers are not yet clear. For the present studies, we focused on the effect of treatments, and the noise from these outliers was identified using the empirical or 68–95–99.7 rule and trimmed or truncated (23) at mean ± two SDs (14, 15).

## Results

Control levels of FITC-d in serum stayed consistent throughout experiments 1–3 with measured serum FITC-d at 0.18 ± 0.01, 0.18 ± 0.02, and 0.17 ± 0.02 μg/mL, respectively (Tables 2–4). Though there was a slight increase noted in experiment 4, at 0.25 ± 0.0 3 μg/mL, significant differences were still noted with FRS and RBD. Levels recovered from the various GIT tissues varied greatly. In experiment 1, serum FITC-d in both treatments was significantly higher than CON, with DSS at 0.29 ± 0.02 μg/mL and FRS at 0.36 ± 0.02 μg/mL, compared to 0.18 ± 0.01 μg/mL (Table 2). For FRS, this difference was repeated in experiment 2 with serum FITC-d measured at 0.37 ± 0.01 and 0.18 ± 0.02 μg/mL in CON, but DSS was not significantly higher at only 0.24 ± 0.02 μg/mL (Table 3). This experiment included an additional group, RBD, which had serum FITC-d levels higher than CON, but no different than DSS, and lower than FRS at 0.28 ± 0.02 μg/mL. In experiments 3 and 4, only FRS and RBD resulted in greater passage of FITC-d to serum with 0.32 ± 0.03 and 0.38 ± 0.02 μg/mL, respectively, compare to 0.25 ± 0.03 μg/mL in the CON group. Neither DSS nor HFD increased FITC-d levels in serum for those experiments.

**Table 2:** **Serum FITC-d and FITC-d retentions in GI tract from experiment 1 using 2-week-old broiler birds**.

Treatments	Serum FITC-d (μg/mL)	FITC-d retention (microgram per gram of tissue)		
		Duodenum	Ileum	Cecum
CON	0.18^c^ ± 0.01	0.27^b^ ± 0.01	0.34^a^ ± 0.02	0.37^b^ ± 0.01
DSS	0.29^b^ ± 0.02	0.28b^ab^ ± 0.01	0.35^a^ ± 0.01	0.41^b^ ± 0.01
FRS	0.36^a^ ± 0.02	0.29^a^ ± 0.01	0.38^a^ ± 0.02	0.46^a^ ± 0.02

*^a–c^Significant (*p* < 0.05) difference within each column*.

*CON, control (basal diet); DSS, dextran sodium sulfate administered at 0.75% in drinking water; FRS, feed restriction for 24 h before processing; *n* = 10/treatment*.

**Table 3:** **Serum FITC-d and FITC-d retentions in GI tract from experiment 2 using 1-week-old leghorn birds**.

Treatments	Serum FITC-d (μg/mL)	FITC-d retention (microgram per gram of tissue)	
		Duodenum	Cecum
CON	0.18^c^ ± 0.02	3.19^b^ ± 0.33	26.16^b^ ± 5.00
DSS	0.24c^bc^ ± 0.02	0.73^c^ ± 0.24	12.64^b^ ± 2.38
FRS	0.37^a^ ± 0.01	5.16^a^ ± 0.63	63.17^a^ ± 13.84
RBD	0.28^b^ ± 0.02	2.51^b^ ± 0.25	25.74^b^ ± 12.42

*^a–c^Significant (*p* < 0.05) difference within each column*.

*CON, control (basal diet); DSS, dextran sodium sulfate administrated at 0.75% in drinking water; FRS, feed restriction for 24 h before processing; RBD, rye-based diet; *n* = 14/treatment*.

**Table 4:** **Serum FITC-d and FITC-d retentions in GI tract from experiments 3 and 4 using 1-week-old broiler birds**.

	Serum (μg/mL)		Duodenum (microgram per gram of tissue)		Cecum (microgram per gram of tissue)	
	Experiment 3	Experiment 4	Experiment 3	Experiment 4	Experiment 3	Experiment 4
CON	0.17^c^ ± 0.02	0.25^c^ ± 0.03	0.73^c^ ± 0.16	0.48^b^ ± 0.12	22.36^b^ ± 6.47	7.55^b^ ± 1.61
DSS	0.26c^bc^ ± 0.04	0.28c^bc^ ± 0.02	1.25c^bc^ ± 0.11	0.70b^ab^ ± 0.06	13.95^b^ ± 3.73	8.65^b^ ± 1.66
HFD	0.29bc^abc^ ± 0.02	0.29c^bc^ ± 0.01	1.35c^bc^ ± 0.18	0.77b^ab^ ± 0.18	9.68^b^ ± 1.48	5.83^b^ ± 1.78
FRS	0.46^a^ ± 0.10	0.32^b^ ± 0.03	2.13^a^ ± 0.22	1.15^a^ ± 0.21	49.69^a^ ± 16.42	37.33^a^ ± 11.17
RBD	0.44b^ab^ ± 0.08	0.38^a^ ± 0.02	1.63b^ab^ ± 0.32	1.05^a^ ± 0.12	4.71^b^ ± 0.90	2.59^b^ ± 0.45

*^a–c^Significant (*p* < 0.05) difference within each column*.

*CON, control (basal diet); DSS, dextran sodium sulfate administrated at 0.75% in drinking water; HFD, high fat diet; FRS, feed restriction for 24 h before processing; RBD, rye-based diet; *n* = 15/treatment*.

Retention of FITC-d in GIT tissue was measured in duodenum, ileum, and cecum in experiment 1 and duodenum and cecum in the other experiments. Differences in retention were noted between FRS and CON groups for duodenum and cecum, but not ileum in the first experiment, though the level of change was not likely biologically significant (Table 2). In experiment 2, FRS resulted in higher retention of FITC-d in both duodenal and cecal tissue with levels measured at 5.16^a^ ± 0.63 and 63.17^a^ ± 13.84 μg/g of tissues, respectively, while DSS levels in the duodenum were lower than CON tissue, this decrease is not consistent with other experiments reported here (Table 3). RBD did not result in changes, compared to CON, in retention of FITC-d in either the duodenum or the cecum (Table 3).

Duodenal retention of FITC-d was affected by FRS (2.13 ± 0.22 and 1.15 ± 0.21 μg/g) and RBD (1.63 ± 0.32 and 1.05 ± 0.12 μg/g) in experiments 3 and 4, compared to CON (0.73 ± 0.16 and 0.48 ± 0.12 μg/g; Table 4). Whereas, in the cecum for experiments 3 and 4, only FRS was different from all other groups with FICT-d retained in tissue, which was 49.69 ± 16.42 and 37.33 ± 11.17 μg/g.

## Discussion

### Serum FITC-d Levels

Determination of serum FITC-d to evaluate enteric inflammation has long been established marker in murine models (11–13). Results from these studies, conducted on chickens, using DSS-FITC-d model (14) suggest that DSS could have caused the disruption of tight junctions in GI tract, increased mucosal permeability (13, 24), ultimately resulting in increased serum FITC-d levels. In experiment 1, the DSS and FRS groups showed higher (*p* < 0.05) serum FITC-d levels when compared to the respective CON (Table 2). This result was in accordance with reports from the studies conducted by Kuttappan et al. (14). Furthermore, FRS group had even higher serum FITC-d level when compared to the respective DSS group (Table 1). Kuttappan et al. (14) showed comparable levels of serum FITC-d between the DSS and FRS groups. This could be because of the fact that Kuttappan et al. (14) used an oral gavage with DSS while the present study administered DSS at the level of 0.75% in drinking water. Thus, when DSS was administered as a high dose for a short period of time (14), it could have a greater impact when compared to low dose prolonged period of administration in the present study, though a direct comparison of the two methods was not completed. In addition, Kuttappan et al. (14) used broilers which were 1-week old while the present study used broiler birds which were 2 weeks old. In fact, the results from these two studies suggested that the serum FITC-d could be used as a marker for gut health in birds across different age groups.

In experiment 2, DSS showed elevated serum FITC-d levels when compared to the respective CON group although it was not significant (*p* > 0.05). However, FRS and RBD groups showed significantly (*p* < 0.05) higher serum FITC-d levels when compared to the respective CON group. Kuttappan et al. (14) previously reported that FRS could result in increased serum FITC-d leakage in broilers. Furthermore, van der Hulst et al. (25, 26) reported that starvation in human patients could result in lack of enough glutamine which could lead to increased gut leakage. Additionally, Tellez et al. (20, 27) reported that RBD, in comparison to corn-based diet, resulted in increased serum FITC-d levels and also significantly (*p* < 0.05) higher translocation of enteric bacteria to liver, both in broiler chicks and turkey poults. The present study was completed in leghorns, and these results confirmed that the model for measuring gut health using serum FITC-d is reliable through different species and strains of poultry.

Experiments 3 and 4 compared serum FITC-d in broilers in CON, DSS, HFD, FRS, and RBD (Table 3). Similar to experiment 2, DSS showed elevated serum FITC-d levels with CON although, it was not significantly different. HFD resulted in a trend similar to DSS both in experiments 3 and 4. de Lartigue et al. (21) had reported that HFD in mice could increase intestinal permeability by altering the expression of tight junction proteins, though results in chickens were not as marked as were reported in mice. Consistent with experiment 2, both FRS and RBD showed higher serum FITC-d levels compared to the respective CON. The results from all four experiments in this trial showed that FRS and RBD could cause increased gut leakage in poultry, which are relevant to current poultry practices. FRS, most commonly skip-a-day feeding, is widely used in meat-type poultry breeder stocks to regulate weight gain and maintain fertility. Increased serum FITC-d due to FRS as reported by the present study suggested that FRS in breeders could result in increased gut leakage and could lead to translocation of enteric bacteria to other tissue organs which could result in disease conditions, such as lameness (28). Similarly, attempts to use least cost feed formulation for poultry often includes the incorporation of alternative feed grains, such as wheat and rye in poultry feed, quite often resulting in reduced performance and poor litter conditions (18, 29, 30). Rye contains high levels of NSP, comprised of highly branched arabinoxylans, which are mainly responsible for increased digesta viscosity, reduced activity of digestive enzymes, and reduced intestinal absorption, making birds susceptible to economically significant conditions, such as necrotic enteritis (19, 31, 32). Our laboratory is currently investigating the effect of various probiotics and dietary enzymes in reducing the gut leakage associated with FRS and RBD based on the serum FITC-d marker.

### Retention of FITC-d in Gastrointestinal Tract Tissues

Kuttappan et al. (14) suggested that the disruption of epithelial layer in the GIT could result in increased infiltration of FITC-d in the paracellular space between cells on the mucosal surface. In all experiments, FRS showed significantly higher FITC-d levels in duodenum and cecum compared to the respective CON birds (Tables 2–4). This was in accordance with the results reported by Kuttappan et al. (14). However, Kuttappan et al. (14) also found that administration of DSS as oral gavage resulted in increased retention of FITC-d in duodenum of broiler chickens when compared to CON, although the cecum did not show any significant difference. With a prolonged administration of low dose DSS (0.75% in drinking water) in the present study, FITC-d did not significantly increase (Tables 2–4). Moreover, RBD, which consistently showed elevated serum FITC-d levels, failed to reflect any difference in GIT tissue FITC-d levels with respect to the CON (Tables 3 and 4). These data suggest that the retention of FITC-d could be more complex which depends upon factors, such as rate of GI passage, which could be affected by irritation on GIT wall, and viscosity of diet as in the case of rye diet. Thus, level of FITC-d retained in GIT tissue may not be a direct measurement of gut leakage as compared to serum FITC-d levels.

## Conclusion

The results from present studies showed that FRS and RBD are consistent methods for inducing mucosal leakage that could lead to enteric inflammation in poultry. Serum FITC-d measurement was proven to be a very reliable and non-invasive marker to determine gut leakage in birds across different age and strains. Since the method involves oral administration of FITC-d and subsequent measurement of FITC-d in serum, it could possibly be used in live birds at multiple time points throughout a single experiment. Further studies will be conducted in our laboratory comparing different growth promoters in poultry and their effect on gut leakage under various conditions using serum FITC-d model.

## Conflict of Interest Statement

The authors declare that the research was conducted in the absence of any commercial or financial relationships that could be construed as a potential conflict of interest.
